# Mechanism of Musashi2 affecting radiosensitivity of lung cancer by modulating DNA damage repair

**DOI:** 10.1002/mco2.548

**Published:** 2024-04-21

**Authors:** Hongjin Qu, Xiong Shi, Ying Xu, Hongran Qin, Junshi Li, Shanlin Cai, Jianpeng Zhao, Bingbing Wan, Yanyong Yang, Bailong Li

**Affiliations:** ^1^ Department of Radiation Medicine Faculty of Naval Medicine Naval Medical University Shanghai China; ^2^ Key Laboratory of Systems Biomedicine (Ministry of Education) Shanghai Center for Systems Biomedicine Shanghai Jiao Tong University Shanghai China; ^3^ Shanghai Engineering Research Center of Tooth Restoration and Regeneration Tongji Research Institute of Stomatology Department of Radiology, Stomatological Hospital and Dental School, Tongji University Shanghai China; ^4^ Department of Nuclear Radiation Shanghai Pulmonary Hospital School of Medicine Tongji University Shanghai China

**Keywords:** ataxia telangiectasia mutated and Rad3‐related kinase (ATR), DNA damage repair, lung cancer, Musashi2 (MSI2), radioresistance, radiosensitivity, RNA‐binding motif protein 17 (RBM17)

## Abstract

Identifying new targets for overcoming radioresistance is crucial for improving the efficacy of lung cancer radiotherapy, given that tumor cell resistance is a leading cause of treatment failure. Recent research has spotlighted the significance of Musashi2 (MSI2) in cancer biology. In this study, we first demonstrated that MSI2 plays a key function in regulating the radiosensitivity of lung cancer. The expression of MSI2 is negatively correlated with overall survival in cancer patients, and the knockdown of MSI2 inhibits tumorigenesis and increases radiosensitivity of lung cancer cells. Cellular radiosensitivity, which is closely linked to DNA damage, is influenced by MSI2 interaction with ataxia telangiectasia mutated and Rad3‐related kinase (ATR) and checkpoint kinase 1 (CHK1) post‐irradiation; moreover, knockdown of MSI2 inhibits the ATR‐mediated DNA damage response pathway. RNA‐binding motif protein 17 (RBM17), which is implicated in DNA damage repair, exhibits increased interaction with MSI2 post‐irradiation. We found that knockdown of RBM17 disrupted the interaction between MSI2 and ATR post‐irradiation and increased the radiosensitivity of lung cancer cells. Furthermore, we revealed the potential mechanism of MSI2 recruitment into the nucleus with the assistance of RBM17 to activate ATR to promote radioresistance. This study provides novel insights into the potential application of MSI2 as a new target in lung cancer radiotherapy.

## Graphical abstract

Scientific hypothesis: After radiation exposure, lung cancer cells undergo DNA damage, triggering RBM17 to sense signals. MSI2, assisted by RBM17, translocates into the nucleus, where it interacts with ATR and CHK1, activating downstream pathways. This activation enhances the DNA damage repair. Upon repair completion, MSI2 and RBM17 dissociate from ATR and exit the nucleus, returning to the initial state.

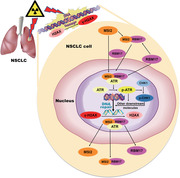

## 1 INTRODUCTION

In recent years, non‐small cell lung cancer (NSCLC) has become one of the major lethal malignancies.^1^, ^2^ Radiotherapy is one of the main treatments for NSCLC, but it is ineffective due to radiation tolerance and poor radiosensitivity.^3^, ^4^ How to increase the radiosensitivity of tumors to improve the effectiveness of radiotherapy for NSCLC has become an important research direction in radiobiology. However, the key problem of specifically increasing the radiosensitivity of NSCLC has not yet been solved, which has led to the unsatisfactory treatment effect of such tumors in clinical practice. Therefore, it is one of the challenges and priorities in radiobiology research at home and abroad to search for new key targets to increase the radiosensitivity of NSCLC, to reveal the molecular mechanism of their effects, and thus to explore new ways to improve the radiotherapy effect of NSCLC.

DNA damage and repair, as the main pathway of the biological effects of ionizing radiation, is not only related to the fate of the irradiated cells but also greatly affects the radiation resistance of tumors. A large number of studies have demonstrated that targeting DNA damage response pathways can improve tumor radiosensitivity, which provides an important research theoretical basis and effective treatment effect for tumor radiotherapy.^5^, ^6^, ^7^, ^8^ With research on tumor radiosensitivity by domestic and foreign scholars, it is promising to explore the radiosensitivity of NSCLC cells from the DNA damage repair pathway. Previous studies have shown that radiation‐resistant tumor cells have stronger DNA damage repair ability, and increasing radiation‐induced DNA damage and inhibiting damage repair are important approaches to study tumor radiosensitization. For example, Ganetespib (an inhibitor of heat shock protein 90 [HSP90]) can cause DNA damage in tumor cells to be unrepaired after radiation and exert radiosensitizing effects, and is currently being used in clinical trials for rectal and esophageal cancers.^9^, ^10^ Inhibition of key DNA damage repair factors such as DNA‐dependent protein kinase catalytic subunit and poly(ADP‐ribose) polymerase 1 (PARP1) have produced some radiosensitizing effects on tumors in both cellular and animal models.^11^, ^12^ These studies suggest that exploring key molecules for tumor radiosensitization from DNA damage repair pathways is an important breakthrough direction that is promising for solving the NSCLC radiosensitization.

RNA‐binding protein Musashi2 (MSI2) has been drawing more and more attention in the field of oncology research in recent years, and it was first reported in hematopoietic stem cells and plays an important role in germ cells and embryonic development.^13^, ^14^ In mammalian species, there are two members of the Musashi family, known as Musashi1 (MSI1) and MSI2. These proteins that share structural similarity, each containing two highly conserved RNA recognition motifs responsible for binding to their respective mRNA targets.^15^, ^16^ Despite their structural similarities, these genes demonstrate unique expression patterns. MSI1 has been extensively researched for its role and presence in stem cells across various adult tissues, including brain, intestine, breast, and colon cancers.^15^, ^17^, ^18^, ^19^ While both MSI1 and MSI2 are found in the brain and testes, MSI2 predominates in hematopoietic tissues and plays a critical role in maintaining the normal function of hematopoietic stem cells and leukemia stem cells in myeloid leukemia.^20^, ^21^, ^22^ Many researchers subsequently found that MSI2 plays an equally important role in the proliferation, invasion, and metastasis of various tumors.^23^ Some researchers screened MSI2 from cell stemness genes by comparing highly invasive lung cancers in NSCLC with less invasive lung cancers and confirmed the highest expression level in metastatic lung cancers, followed by primary lung cancers, and the lowest in normal lung tissues by clinical samples. Thus, MSI2 is highly associated with epithelial‒mesenchymal transition (EMT) in NSCLC, and its regulation of transforming growth factor‐beta (TGF‐β) and inhibition of claudins ultimately promote the invasive metastasis of NSCLC.^22^, ^24^ MSI1, a homologous protein of MSI2, has been reported to regulate radiation‐induced DNA damage by promoting non‐homologous end joining in malignant gliomas, and inhibition of MSI1 increases radiosensitivity of malignant gliomas.^11^ MSI1 can enhance the invasion of glioblastoma multiforme and increase homologous recombinant repair and apoptosis evasion through activation of the DNA damage response pathway, thereby increasing radiation resistance in glioblastoma multiforme, which may serve as a new target for the treatment of this tumor.^25^ What exactly does MSI2 do, and does it also play a radiation resistance role in NSCLC? And does it work like MSI1 through the DNA damage repair pathway? No studies have been reported.

Our current study revealed that MSI2 is vital for regulating the radiosensitivity of lung cancer cells. Its expression level was negatively correlated with the survival rate of lung cancer patients, and knockdown of MSI2 had a significant radiosensitizing effect on lung cancer cells, suggesting that it has a significant impact on lung cancer radiosensitivity and has good potential to overcome lung cancer radioresistance. The interaction of RNA‐binding motif protein 17 (RBM17) with MSI2 was identified through immunoprecipitation‐mass spectrometry (IP‐MS), revealing its involvement in DNA damage repair and tumor development.^26^, ^27^, ^28^, ^29^ We found that MSI2 and RBM17 started to enter the nucleus at 0.5 h after irradiation and gradually increased, and their interaction was enhanced. MSI2 entering the nucleus was required with the assistance of RBM17, as knockdown of RBM17 inhibited the entry of MSI2 into the nucleus and the interaction between MSI2 and ataxia telangiectasia mutated and Rad3‐related kinase (ATR). MSI2 and RBM17 entered the nucleus at their highest levels at 8 h after irradiation and interacted with ATR, which activated ATR and its downstream checkpoint kinase 1 (CHK1) phosphorylation, and both exited the nucleus in large amounts at 24 h. Knockdown of MSI2 significantly reduced the phosphorylation level of ATR after irradiation. Therefore, we propose the hypothesis that MSI2 may interact with ATR with the assistance of RBM17 in the DNA damage repair pathway and change its subcellular localization, thus affecting DNA damage repair and ultimately changing the radiosensitivity of cells.

## 2 RESULTS

### 2.1 High expression of MSI2 in lung cancer was negatively correlated with patient prognosis

**FIGURE 1:**
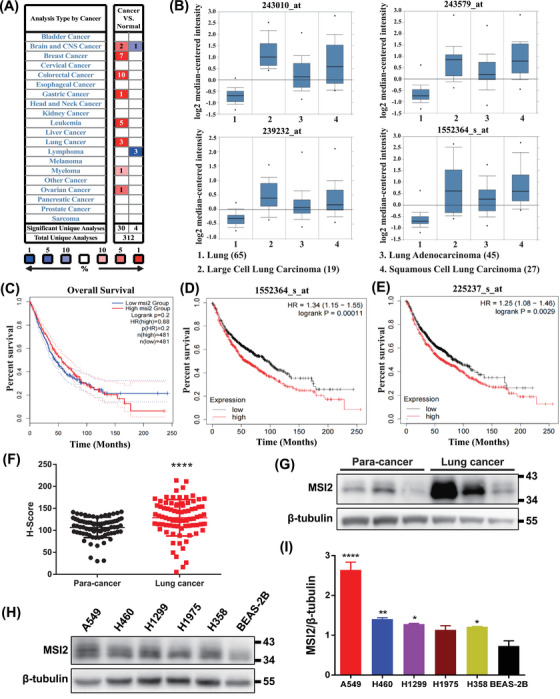
Musashi2 (MSI2) was highly expressed in lung cancer and negatively correlated with patient survival. (A) The transcript levels of MSI2 in different tumors compared to normal tissue, both red and blue represent statistically significant differences, with darker color representing greater difference. Red represents tumors with high mRNA expression relative to normal tissue and blue being the opposite. (B) The comparison of MSI2 at transcript levels in normal lung tissue and non‐small cell lung cancer in large cell lung cancer, adenocarcinoma, and squamous carcinoma under four different probes. (C) The overall survival of gene MSI2 in lung adenocarcinoma (LUAD) and lung squamous cell carcinoma (LUSC) from multiple datasets. (D and E) Kaplan‒Meier survival analysis of MSI2 mRNA expression levels and lung cancer prognosis under two different probes. Bioinformatics analysis data of (A) and (B) from ONCOMINE; (C) from GEPIA 2; (D) and (E) from Kaplan‒Meier plotter. (F) Immunohistochemistry score statistics of tissue chip immunohistochemistry detection of lung cancer samples from 80 lung cancer patients (^****^ *p* < 0.0001). (G) Western blotting tests of lung cancer tissue sample and corresponding paraneoplastic tissue sample. Lanes 1 and 4 are the same case. Lanes 2 and 5 are the same case. Lanes 3 and 6 are the same case. (H) Protein expression of MSI2 in each non‐small cell lung cancer cell lines and normal lung bronchial epithelial cells. (I) Statistical analysis of grayscale values of (E) (^*^ *p* < 0.05, ^**^ *p* < 0.01, ^****^ *p* < 0.0001).

Initially, we employed bioinformatic methods to analyze the function of MSI2. The ONCOMINE analysis indicated significant upregulation of MSI2 in lung cancer compared to adjacent normal tissues, as well as in breast cancer, colon cancer, gastric cancer, and leukemia (Figure 1A). Compared to normal lung tissue, the mRNA expression levels of MSI2 were notably elevated in large‐cell lung cancer, adenocarcinoma, and squamous carcinoma (all NSCLC), as confirmed by four different probes (Figure 1B). Subsequently, GEPIA 2 analysis (http://gepia2.cancer‐pku.cn) further confirmed the higher expression of MSI2 in lung cancer, particularly in lung adenocarcinoma and lung squamous cell carcinoma (Figure S1A,B). Elevated MSI2 expression correlated with poor overall survival (Figure 1C). To delve deeper, we conducted Kaplan‒Meier survival analysis of MSI2 mRNA expression levels and lung cancer prognosis using Kaplan‒Meier plotter. The results indicated that patients with high MSI2 mRNA expression levels exhibited lower survival rates, with statistically significant differences in both probe groups (Figure 1D,E). This finding suggested that higher MSI2 expression is associated with a worse prognosis in lung cancer.

For further validation, we performed immunohistochemical detection on tissue microarrays from 80 lung cancer patients and corresponding normal lung tissues adjacent to the cancer (Figure S1C,D). *H*‐score and Western blotting analyses revealed a significantly higher expression level of MSI2 in lung cancer tissues than in their adjacent normal lung tissues (Figure 1F,G). Western blotting tests also confirmed the elevated expression of MSI2 in NSCLC cell lines, especially in A549, compared to normal lung cells (Figure 1H,I). These findings collectively affirm the high expression of MSI2 in clinical lung cancer tissues and NSCLC cell lines.

### 2.2 Knockdown of MSI2 inhibits tumorigenesis and increases radiosensitivity of lung cancer cells

**FIGURE 2:**
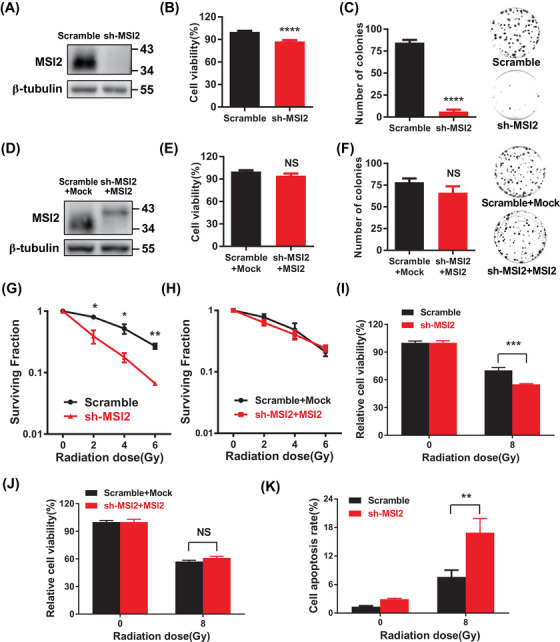
Knockdown of Musashi2 (MSI2) inhibits tumorigenesis and increases radiosensitivity of lung cancer cells. (A) Protein validation of knockdown of MSI2 (sh‐MSI2) in A549 cells. (B) Changes in A549 cells proliferation after knockdown of MSI2 (^****^ *p* < 0.0001). (C) Clone formation of A549 cells after knockdown of MSI2. (D) Protein validation of partial restoration of MSI2 expression (fusion with label 3×FLAG) after knockdown of MSI2 (sh‐MSI2 + MSI2). (E) Effect of partial restoration of MSI2 expression after knockdown of MSI2 on cell proliferation. (F) Effect of partial restoration of MSI2 expression on clone formation after knockdown of MSI2. (G) Data statistics after knockdown of MSI2 at different doses of irradiation (^*^ *p* < 0.05, ^**^ *p* < 0.01). (H) Statistical analysis of RESCUE MSI2 expression on the clone formation in A549 cells after irradiation. (I) Proliferation level of 8 Gy irradiated A549 cells after knockdown of MSI2 (^***^ *p* < 0.001). (J) Effect of RESCUE MSI2 expression on the proliferation level of A549 cells after irradiation. (K) Statistical analysis of the apoptosis levels in A549 cells irradiated with 8 Gy after MSI2 knockdown (^**^ *p* < 0.01).

To further explore the role of MSI2 in lung cancer, we found that knockdown of MSI2 significantly decreased the proliferation level of A549 cells and reduced the clonogenic ability (Figure 2A‒C), which indicated that MSI2 plays an important role in the proliferation of lung cancer cells. In contrast, the cell proliferation level was notably increased, and the clonogenic ability was also obviously enhanced in the MSI2 rescue assay (Figure 2D‒F). These results were also verified by nude mouse tumor‐bearing experiments in vivo (Figure S2A,B). These data show that the absence of MSI2 inhibits the proliferation and cloning ability of lung cancer cells and knockdown of MSI2 inhibits the tumorigenesis of lung cancer cells.

To explore the potential influence of MSI2 on the radiosensitivity of lung cancer cells, we initially assessed the response of MSI2 to radiation. Building on our previous experience with irradiating alveolar epithelial cells, we exposed A549 cells to a single dose of 8 Gy and observed a substantial increase in MSI2 protein levels at 8 h post‐irradiation (Figure S2C).^30^ Subsequently, we confirmed a significant elevation in MSI2 protein levels at 8 h after 8 Gy irradiation (Figure S2D). Next, we performed a standard clonogenic assay in A549 cells, a well‐established method for evaluating radiosensitivity. Our findings demonstrated that the reduction in MSI2 expression significantly impeded the clonogenic survival of lung cancer cells post‐irradiation, with the suppressive effect becoming more pronounced with increasing radiation doses (Figures S2E and 2G). Conversely, clonogenic survival was restored upon MSI2 restoration (Figure 2H). Furthermore, the knockdown of MSI2 markedly diminished the relative proliferation of cells post‐irradiation, and this effect was reversed in MSI2 rescue experiments (Figure 2I,J). Similar outcomes were observed in H460 and H1299 cells (Figure S2F,G). Additionally, MSI2 knockdown notably increased apoptosis in A549 cells following irradiation (Figures S2H and 2K). These findings suggest that the downregulation of MSI2 in lung cancer cells could enhance their sensitivity to radiation. In summary, MSI2 appears to play a significant role in the tumorigenesis and radiosensitivity of lung cancer cells.

### 2.3 Knockdown of MSI2 inhibits the ATR‐mediated DNA damage response pathway

**FIGURE 3:**
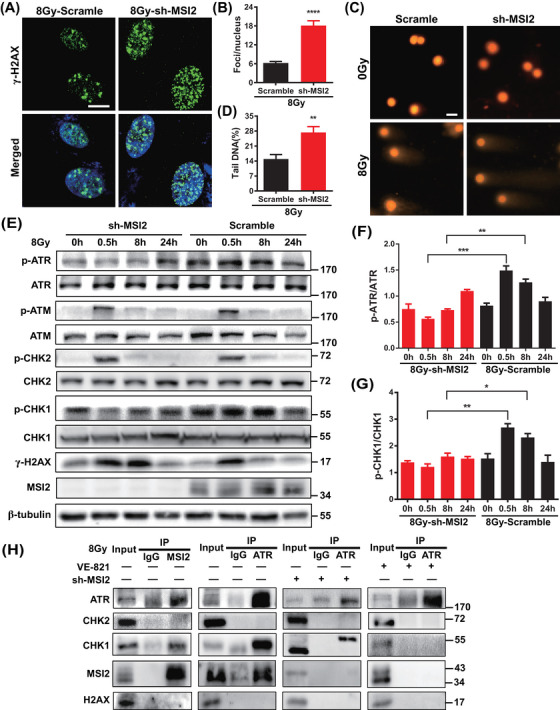
Knockdown of Musashi2 (MSI2) inhibits the ataxia telangiectasia mutated and Rad3‐related kinase (ATR)‐mediated DNA damage response pathway. (A) Representative images of gamma‐H2AX (γ‐H2AX) foci in A549 cells at 24 h after 8 Gy irradiation (scale bar, 10 µm). (B) Data statistics of γ‐H2AX foci in (A) (^****^ *p* < 0.0001). (C) Representative comet electrophoresis assay at 8 h after 8 Gy irradiation (scale bar, 10 µm). (D) DNA trailing data statistics of (C) (^**^ *p* < 0.01). (E) Protein expression level of each molecule in the DNA damage response pathway in A549 cells at 0, 0.5, 8, and 24 h after 8 Gy irradiation. (F and G) Statistical analysis of grayscale values of (E) (^*^ *p* < 0.05, ^**^ *p* < 0.01, ^***^ *p* < 0.001). (H) Immunoprecipitation assay of MSI2 and ATR in A549 cells 8 h after 8 Gy irradiation. VE‐821 is a specific inhibitor of ATR.

Cellular sensitivity to radiation is closely associated with the level of DNA damage. To further elucidate how MSI2 influences the radiation sensitivity of lung cancer cells, we investigated the correlation between MSI2 and DNA damage following irradiation. We assessed the extent of DNA damage by measuring the number of gamma‐H2AX (γ‐H2AX) foci and the length of DNA comet tails post‐irradiation. As depicted in Figures 3A,B and S3A, the depletion of MSI2 markedly increased the number of γ‐H2AX foci in A549 cells after irradiation. Furthermore, Figure 3C,D demonstrates that MSI2 knockdown augmented the length of DNA comet tails in A549 cells following irradiation. These findings suggest that the downregulation of MSI2 enhances radiation‐induced DNA damage.

Next, we aimed to investigate whether MSI2 influences DNA damage through the DNA damage response pathway and, if so, which specific pathway is involved. Subsequently, using Western blot analysis (Figure 3E), we observed that the depletion of MSI2 increased the levels of the DNA damage marker γ‐H2AX in A549 cells, particularly at 8 h post‐irradiation. This finding further supports the idea that MSI2 downregulation augments radiation‐induced DNA damage. Concurrently, MSI2 knockdown notably decreased the levels of p‐ATR and p‐CHK1, especially at 0.5 and 8 h post‐irradiation, without significantly affecting the expression of the phosphorylation of ataxia telangiectasia mutated kinase (p‐ATM) and the phosphorylation of checkpoint kinase 2 (p‐CHK2) (Figure 3F,G). These results suggest that MSI2 knockdown may enhance radiation‐induced DNA damage by potentially suppressing the ATR‐mediated DNA damage response pathway.

We delved deeper into understanding how MSI2 impacts the ATR‒CHK1 pathway. Using immunoprecipitation (IP) experiments (Figures S3B and 3H), we observed that MSI2 interacts with ATR and CHK1 following irradiation. Furthermore, knocking down MSI2 suppressed these interactions, while the ATR inhibitor VE‐821 notably disrupted them. Interestingly, we noticed an upward shift in the detected bands during Western blot analysis in post‐irradiation IP experiments using an ATR antibody. We hypothesized that this phenomenon could be attributed to certain modifications of the captured proteins, such as phosphorylation modifications. However, further experiments are necessary to explore this possibility. In summary, we suggested that MSI2 may impact the radiation‐induced DNA damage response and, consequently, the radiation sensitivity of lung cancer cells through its interaction with ATR.

### 2.4 Knockdown of RBM17 disrupts the interaction between MSI2 and ATR post‐irradiation and increases the radiosensitivity of lung cancer cells

**FIGURE 4:**
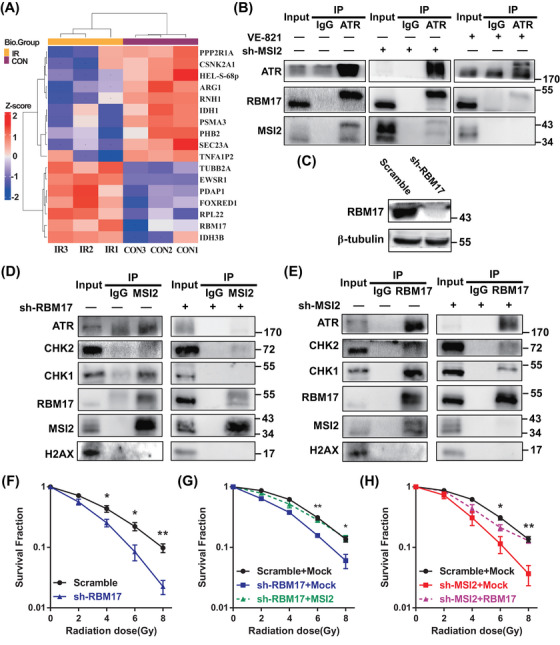
Knockdown of RNA‐binding motif protein 17 (RBM17) disrupts the interaction between Musashi2 (MSI2) and ataxia telangiectasia mutated and Rad3‐related kinase (ATR) post‐irradiation and increases the radiosensitivity of lung cancer cells. (A) Heat map of immunoprecipitation‐mass spectrometry (IP‐MS) assay of partial differential protein hierarchical clustering analysis. Top color bar represents the sample grouping and the bottom is the corresponding sample name. CON represents the control group without irradiation and IR represents the irradiated group with 8 Gy irradiation. The left legend *Z*‐score represents the relative expression level of proteins by color, in which red represents high expression and blue represents low expression. The darker color represents the more obvious difference. (B) Immunoprecipitation assay of ATR at 8 h after 8 Gy irradiation of A549 cells. VE‐821 is a specific inhibitor of ATR. (C) Protein validation of RBM17 knockdown (sh‐RBM17). (D) Immunoprecipitation assay of MSI2 at 8 h after 8 Gy irradiation of A549 cells. (E) Immunoprecipitation assay of RBM17 at 8 h after 8 Gy irradiation of A549 cells. (F) Statistics of clone formation after RBM17 knockdown irradiated with different doses (^*^ *p* < 0.05, ^**^ *p* < 0.01). (G) Statistics of clone formation after RBM17 knockdown and knockdown of RBM17 with overexpressing MSI2 (sh‐RBM17 + MSI2) (^*^ *p* < 0.05, ^**^ *p* < 0.01). (H) Statistics of clone formation after MSI2 knockdown and MSI2 knockdown with overexpressing RBM17 (sh‐MSI2 + RBM17) (^*^ *p* < 0.05, ^**^ *p* < 0.01).

As an RNA‐binding protein, MSI2, has predominantly been studied for its interactions with RNA, with limited research on its protein‒protein interactions. Earlier, we observed the interaction of MSI2 with ATR and CHK1 proteins post‐irradiation (Figures S3B and 3H), prompting our hypothesis that proteins exhibiting significant changes in their interaction strength with MSI2 before and after irradiation might provide valuable insights into the mechanisms underlying MSI2's influence on the radiation‐induced DNA damage response. Consequently, we conducted experiments utilizing IP‐MS to explore potential protein interactors with MSI2 in both pre‐ and post‐irradiation contexts. We divided A549 cells into two groups, one for control (CON) group (no irradiation) and one for irradiated (IR) group (8 Gy irradiation). The proteins interacting with MSI2 were extracted by IP using IP‐MS, which detected 6564 peptides and identified 1362 proteins. The number of quantifiable proteins was 1215, including 216 differential proteins and 17 molecules with significant differences such as RBM17 (Figures 4A and S4A). RBM17, also known as splicing factor 45, is a dual‐functional RNA‐binding protein known to play roles in splicing and DNA damage repair. It is involved in the alternative splicing of the apoptosis regulatory gene FAS, overexpressed in breast, ovarian, and prostate cancers, and contributes to tumor drug resistance.^26^, ^27^, ^28^, ^29^ Given its relevance to DNA damage repair and tumor development, similar to our study on MSI2, we found it intriguing and chose to delve deeper into understanding the relationship, functions, and mechanisms between MSI2 and RBM17. We further confirmed that RBM17 interacted strongly with MSI2 after irradiation through the Co‐IP assay (Figure S4B,C).

Then, we used IP to further clarify the relationship between ATR, MSI2, and RBM17 (Figures S3B and 4B). We found ATR interacted with RBM17 and MIS2 after irradiation. Knocking down MSI2 did not affect the interaction between ATR and RBM17. However, the use of the ATR inhibitor VE‐821 significantly disrupted the interactions between ATR and both MSI2 and RBM17. Then, we used shRNA to knockdown RBM17 (Figure 4C). Knockdown of RBM17 significantly inhibited the interaction between MSI2 and ATR (Figure 4D), while knockdown of MSI2 did not affect the interaction between RBM17 and ATR (Figure 4E), indicating that RBM17 may play a key role in the interaction of MSI2 and ATR after irradiation.

We found that knockdown of RBM17 significantly reduced the clone formation rates of 4, 6, and 8 Gy, indicating that knockdown of RBM17 has a radiosensitizing effect on A549 cells (Figure 4F). We have previously shown that knockdown of MSI2 also has a radiosensitizing effect, so what is the relationship between MSI2 and RBM17 in radiosensitization studies? We next transfected the MSI2 overexpression plasmid in the stable transfected cell line with knockdown RBM17 and the RBM17 overexpression plasmid in the stable transfected cell line with knockdown MSI2 for clone formation assays. Compared to the sh‐RBM17 + Mock group, the clone formation rate of the sh‐RBM17 + MSI2 group was significantly higher at 6 and 8 Gy irradiation, indicating that MSI2 was able to partially restore the clone formation ability after knocking down RBM17 (Figure 4G). Similarly, the clone formation rate of sh‐MSI2 + RBM17 group was significantly higher than the sh‐MSI2 + Mock group at 6 and 8 Gy irradiation, indicating that MSI2 was also able to partially restore the clone formation ability after knocking down MSI2 (Figure 4H). These data indicate a mutual promotion between MSI2 and RBM17 in promoting radioresistance in lung cancer.

### 2.5 Effect of RBM17 and ATR on MSI2 nuclei entry and exit

**FIGURE 5:**
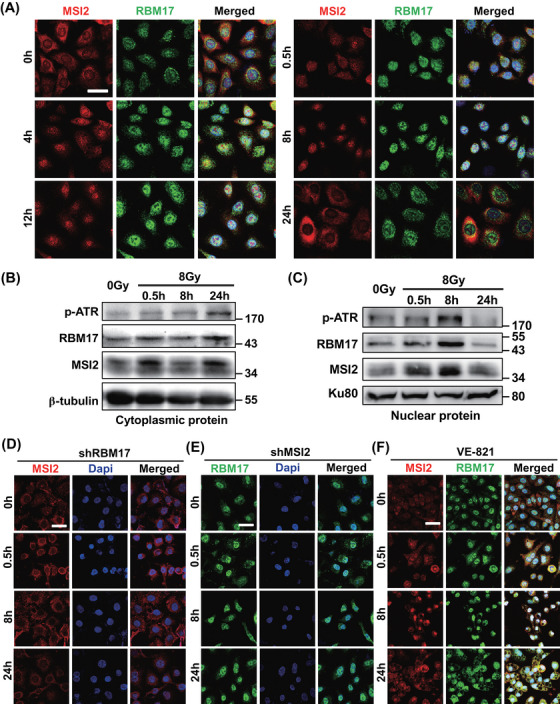
Effect of RNA‐binding motif protein 17 (RBM17) and ataxia telangiectasia mutated and Rad3‐related kinase (ATR) on Musashi2 (MSI2) entry and exit the nucleus. (A) Confocal immunofluorescence photographs of MSI2 and RBM17 at 0, 0.5, 4, 8, 12, and 24 h after irradiation (scale bar, 20 µm). (B) Cytoplasmic protein detection at 0, 0.5, 8, and 24 h after 8 Gy irradiation. (C) Nuclear protein detection at 0, 0.5, 8, and 24 h after 8 Gy irradiation. (D) Confocal immunofluorescence photographs of MSI2 at each time point of 8 Gy irradiation after knockdown of RBM17 in A549 cells (scale bar, 20 µm). (E) Confocal immunofluorescence photographs of RBM17 at each time point of 8 Gy irradiation after knockdown of MSI2 in A549 cells (scale bar, 20 µm). (F) Confocal immunofluorescence photographs of MSI2 and RBM17 at each time point of 8 Gy irradiation after adding ATR inhibitor VE‐821 (scale bar, 20 µm).

To further elucidate the mechanism by which MSI2 and RBM17 induce radioresistance in lung cancer cells, we performed immunofluorescence detection of MSI2 and RBM17 in A549 cells at 0, 0.5, 4, 8, 12, and 24 h post‐irradiation with 8 Gy. Confocal microscopy was employed for image acquisition. As Figure 5A shows, both MSI2 and RBM17 started to enter the nucleus at 0.5 h after irradiation and reached their peak at 8 h. They gradually started to exit the nucleus at 12 h and almost completely got out at 24 h. To further confirm the results of immunofluorescence of RBM17 and MSI2 in A549 cells after irradiation, we extracted cytoplasmic and nuclear protein from cells at different time points after irradiation and examined the protein changes inside and outside the nucleus of MSI2 and RBM17 by Western blotting. In the cytoplasm, MSI2 and RBM17 increased at 0.5 h, decreased at 8 h, and increased again at 24 h, which is consistent with the changes of confocal immunofluorescence assay (Figure 5B). In the nucleus, MSI2 and RBM17 also increased at 0.5 h, and increased more obviously at 8 h, but decreased at 24 h, which is consistent with the changes of confocal immunofluorescence assay (Figure 5C). The protein expression level of p‐ATR, representing the activated state of ATR, changed in the cytoplasm and nucleus after irradiation. In the cytoplasm, its expression increased at 0.5 h, remained constant at 8 h, and exhibited a significant increase at 24 h (Figure 5B). In the nucleus, there was a significant increase at 8 h followed by a decrease at 24 h (Figure 5C). These findings suggested that ATR phosphorylation activation peaks at 8 h and diminishes at 24 h, correlating with the dynamic entry and exit of MSI2 and RBM17.

To further validate the IP results, we explored the effects of RBM17 knockdown, MSI2 knockdown, and ATR inhibitors on MSI2 and RBM17 entry into the nucleus by confocal immunofluorescence. As shown in Figure 5D, MSI2 failed to enter the nucleus at 8 h in A549 cells (RBM17 knockdown) irradiated with 8 Gy, in contrast to the results in Figure 5A, indicating that RBM17 plays a key role in MSI2 entering the nucleus after irradiation. Knockdown of MSI2 made RBM17 stay in the nucleus all the time (Figure 5E). Entry of MSI2 and RBM17 into the nucleus after irradiation was not affected by VE‐821, but exit from the nucleus at 24 h was inhibited (Figure 5F). These data suggested that MSI2 requires the assistance of RBM17 to enter the cell nucleus, and inhibiting ATR activity prevents the nuclear exit of MSI2.

Considering the preceding results, we proposed that after radiation, the interaction between MSI2 and RBM17 intensifies, leading to a substantial entry into the cell nucleus with RBM17 assistance. Subsequently, MSI2 interacts with ATR, activating ATR and its downstream pathways, thereby promoting DNA damage repair. Knocking down MSI2 results in a reduction of nuclear entry and interaction with ATR, leading to decreased ATR activation and impaired DNA damage repair. Consequently, RBM17, aiming to recruit more MSI2 into the nucleus for ATR activation, persists in the nucleus. When VE‐821 inhibits ATR activation, normal DNA damage repair is disrupted, prompting MSI2 and RBM17 to remain in the nucleus reflexively, facilitating interaction with ATR and subsequent activation.

### 2.6 Enhancing the radiosensitivity of lung cancer through knockdown of MSI2 or RBM17 in vivo

**FIGURE 6:**
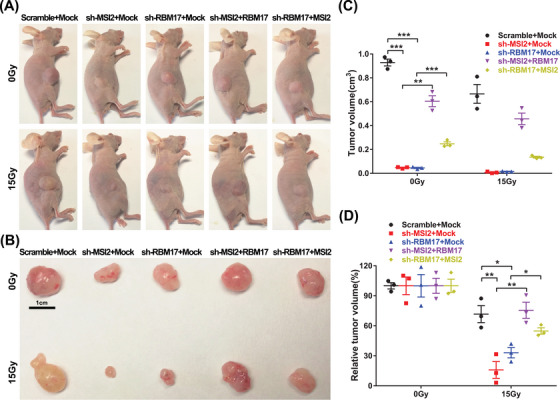
In vivo tumor loading experiments. (A) Photographs of representative nude mice of each group. (B) Photographs of representative tumor bulk specimens of each group in nude mice. (C) Data statistics of tumor volume after 0 and 15 Gy irradiation in each group (*n* = 3, ^**^ *p* < 0.01, ^***^ *p* < 0.001). (D) Data statistics of relative tumor volume in each group based on the tumor volume of 0 Gy in this group (*n* = 3, ^*^ *p* < 0.05, ^**^ *p* < 0.01).

Based on the experimental results presented above, we posit that MSI2 enhances DNA damage repair through interactions with RBM17 and ATR, thereby increasing the radiation resistance of lung cancer cells. To validate the role of MSI2 and RBM17 in radioresistance, we constructed stable transgenic cell lines with knockdown of MSI2 and RBM17 and overexpressed another molecule in the knockdown cell line, and nude mice were tumor‐loaded subcutaneously. After 1 month, half the number of nude mice in each group were randomly selected for localized 15 Gy irradiation of tumors, and tumor size was measured and tumor tissues were removed from nude mice by execution after 0.5 month. For the non‐irradiated tumors, we found that knockdown of MSI2 and RBM17 could significantly inhibit tumor growth. Compensating RBM17 in knockdown MSI2 and MSI2 in knockdown RBM17 resulted in significantly larger tumors compared to control, indicating that RBM17 partially restored the inhibition of tumors in knockdown MSI2 and MSI2 partially restored the inhibition of tumors in knockdown RBM17 on tumor suppression. The tumor volume was significantly reduced after local irradiation of 15 Gy, and the irradiation significantly inhibited the tumor growth, which confirmed the importance of radiotherapy as the main treatment for NSCLC (Figure 6A,B). All the images from three independent experiments are shown in Figure S5.

Subsequently, the tumor size after irradiation was compared with the tumor size without irradiation in this group, then we performed statistical analysis to exclude the interference of inconsistent tumor growth size, and only compared the effect of irradiation factors on tumor size in each group. We found that knockdown of either MSI2 or RBM17 had significant radiosensitizing effects, and MSI2 partially reversed the effect of knockdown of RBM17 on tumor radiosensitivity, while RBM17 significantly reversed the effect of knockdown of MSI2 on tumor sensitivity (Figure 6C,D).

In conclusion, MSI2 and RBM17 played a radioresistant role in lung cancer radiotherapy, and RBM17 played a more important role for MSI2 in lung cancer radioresistance, which is consistent with the mechanism that MSI2 promotes radioresistance by activating ATR through the assistance of RBM17 into the nucleus.

## 3 DISCUSSION

**FIGURE 7:**
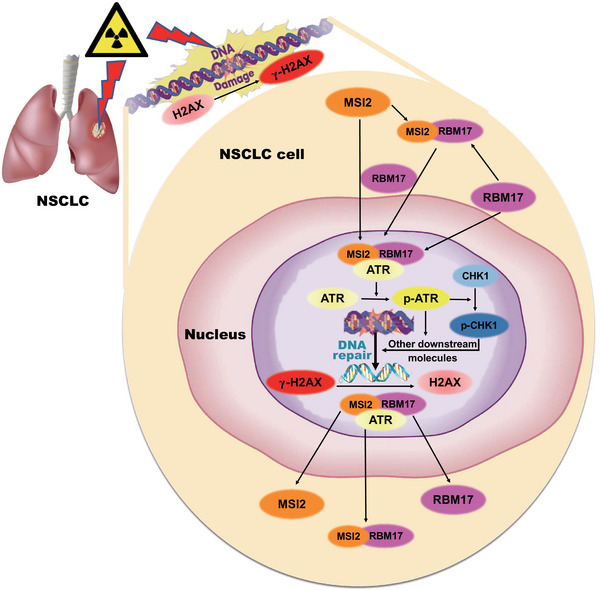
Scientific hypothesis: when lung cancer cells undergo DNA damage after radiation exposure and emit certain signals, RNA‐binding motif protein 17 (RBM17) may sense these signals, translocate into the nucleus, facilitate the entry of Musashi2 (MSI2), and subsequently interact with ataxia telangiectasia mutated and Rad3‐related kinase (ATR), activating ATR along with checkpoint kinase 1 (CHK1) and other downstream molecules. This activation promotes the DNA damage response pathway, facilitating DNA damage repair. When DNA damage repair is completed, MSI2 and RBM17 receive certain signals, dissociate from ATR, and exit the nucleus to return to the initial state.

There are three main conventional treatments for tumors: surgical treatment, radiotherapy, and chemotherapy, which can be used alone or in combination with each other. Radiotherapy is widely used as a non‐invasive treatment with fewer side effects. Chemotherapy is used as a supplement to surgery and radiotherapy to treat metastatic and multiple tumors and is often used in combination with radiotherapy. It has been reported that 80% of lung cancers are NSCLC with poor prognosis, and radiotherapy is an indispensable tool for its difficult clinical treatment.^31^ However, the greatest challenge of radiotherapy in clinic is the damage to normal tissues and radiation resistance. In this study, we proposed a hypothesis on the mechanism of MSI2 in lung cancer radiosensitivity (Figure 7). When lung cancer cells undergo DNA damage after radiation exposure and emit certain signals, RBM17 may sense these signals, translocate into the nucleus, facilitate the entry of MSI2, and subsequently interact with ATR, activating ATR along with CHK1 and other downstream molecules. This activation promotes the DNA damage response pathway, facilitating DNA damage repair. When DNA damage repair is completed, MSI2 and RBM17 receive certain signals, dissociate from ATR, and exit the nucleus to return to the initial state. When MSI2 is knocked down or inhibited in lung cancer cells, there is not enough MSI2 to enter the nucleus after DNA damage, so it cannot interact with RBM17 and ATR to activate ATR, which will not successfully complete DNA damage repair and increase tumor cell death. The result is that the tumor cells will not be able to repair DNA damage, which will increase cell death and thus exert radiosensitizing effects.

MSI2 is vital for the maintenance of cell stemness, and subsequently many researchers found that it plays an equally important role in the proliferation, invasion, and metastasis of various tumors. Some researchers screened MSI2 from cell stemness genes by comparing highly invasive lung cancers with less invasive lung cancers in NSCLC, and confirmed the highest expression level in metastatic lung cancers, followed by primary lung cancers, and lowest in normal lung tissues by clinical samples, thus suggesting that MSI2 is highly correlated with EMT in NSCLC by regulating TGF‐β and inhibiting claudins ultimately promote the invasive metastasis of NSCLC. Based on these studies, we first found that MSI2 was expressed at higher levels than normal tissues in many tumors, such as lung, breast, and colon cancers, and especially in all types of NSCLC than in normal lung tissues by bioinformatics analysis. We made tissue microarrays of human clinical lung cancer tissues and paraneoplastic tissues and confirmed the expression level of MSI2 in lung cancer tissues was higher than that in normal lung tissues by immunohistochemistry. We further confirmed that MSI2 plays an important role in the development of lung cancer by knocking down MSI2 and RESCUE MSI2 to detect cell clone formation and proliferation levels. Knockdown MSI2 had a radiosensitizing effect on lung cancer and MSI2 played a radioresistant role in lung cancer radiotherapy. According to our knowledge, MSI2 has not been reported in the study of radiosensitivity in NSCLC. MSI2 plays a role in promoting tumor invasion and metastasis, consistent with the findings described previously.

We are interested in the radioresistance of MSI2, and knocking down the radiosensitizing effect of MSI2 on lung cancer, either by gene therapy or chemical inhibitor development, has practical clinical applications for improving the efficacy of radiotherapy in NSCLC. In order to better serve the clinical needs, pure effect studies are not enough, but precise mechanism studies are essential. Most of the promising studies on sensitizers are focused on the DNA damage response pathway. Ganetespib, an inhibitor of HSP90, has been shown to increase radiosensitivity in cellular models. Hsp90 is involved in correcting the folding of a range of related proteins, directly involved in regulating the folding of 725 proteins, including the key proteins of the DNA damage response pathway. Therefore, inhibition of Hsp90 directly leads to the inability of key proteins to fold correctly and thus to play a role in DNA damage repair, which ultimately leads to the inability of DNA damage in tumor cells to be repaired after radiation. As a result, the DNA damage in tumor cells cannot be repaired after radiation and exerts radiosensitizing effects.^10^, ^32^ Because of its low hepatotoxicity and nephrotoxicity and high safety, Ganetespib is currently is being used in clinical trials for rectal and esophageal cancers, and is one of the most promising inhibitors of Hsp90 for clinical use as a radiosensitizer.^9^ Olaparib, an inhibitor of PARP, has been used as an inhibitor in the treatment of BRCA (breast cancer susceptibility genes)‐mutant ovarian and breast cancers. It is also expected to be used as a radiosensitizer in clinical trials in combination with radiotherapy for NSCLC and breast cancer.^33^ The DNA damage response pathway plays an important role in the study of tumor radiotherapy as a breakthrough in radiotherapy sensitivity research.

Two important indicators of DNA damage, the number of γ‐H2AX foci and the length of DNA tails after irradiation, have been widely used in radiation studies.^8^ We examined the changes of DNA damage after knockdown of MSI2 by these two indicators to investigate the relationship between MSI2 and DNA damage response pathway. Knockdown of MSI2 not only significantly increased the number of γ‐H2AX foci after irradiation, but also significantly increased the length of DNA tails in lung cancer cells, which indicated that knockdown of MSI2 increased the degree of DNA damage, or inhibited the DNA damage repair. We then found that MSI2 inhibited the activation of ATR and its downstream major effector CHK1, but had no significant effect on the activation of ATM and its downstream major effector CHK2, suggesting that MSI2 plays a role in the ATR‐mediated DNA damage repair. Furthermore, the IP‐MS analysis of MSI2 led to the identification of RBM17, a protein that interacts with MSI2. RBM17 was initially identified for its role in DNA splicing and was later suggested to play a role in DNA damage repair, and was also reported to be associated with tumorigenesis and invasive metastasis.^26^, ^27^, ^28^, ^34^ However, studies on RBM17 in tumor radiosensitivity have not been reported yet. We found that MSI2 interacts with RBM17 in an enhanced manner after irradiation, and the relationship between them deserves further investigation.

We first found that knockdown of RBM17 produced radiosensitization of lung cancer cells. RBM17 and MSI2 may synergistically promote radioresistance in lung cancer. We observed that RBM17 and MSI2 entered the nucleus sequentially after irradiation (RBM17 before MSI2), with mostly entry at 8 h and exit at 24 h. The same results were also obtained by Western blotting analysis of cytoplasmic and nuclear proteins. By IP, we found that the three proteins interact with each other and may form a complex after irradiation. By using inhibitors, we found that as long as RBM17 was knocked down, MSI2 would not be able to interact with ATR after irradiation. The inhibition of ATR activation has no effect on the nuclear entry of MSI2 and RBM17 after irradiation, but it inhibits their nuclear exit. Based on the above results, we have drawn a schematic diagram of MSI2, RBM17, and ATR in lung cancer radiotherapy (Figure 7), which shows that lung cancer cells undergo DNA damage repair after radiation exposure and emit certain signals, RBM17 may first sense the signal and enter the nucleus, while recruiting MSI2 and assisting MSI2 to enter the nucleus. RBM17 may first sense the signal and enter the nucleus, and at the same time, recruit MSI2 and assist MSI2 to enter the nucleus; then, interact with ATR to form a complex and activate ATR, and then activate CHK1 and other downstream molecules, thus activating the DNA damage response pathway and promoting DNA damage repair. When DNA damage repair is completed, MSI2 and RBM17 receive certain signals and dissociate from the ATR and exit the nucleus to restore the initial state. When lung cancer cells are irradiated after knocking down MSI2, there is not enough MSI2 to enter the nucleus after DNA damage occurs, and it cannot interact with RBM17 and ATR to activate ATR, resulting in DNA damage repair not proceeding smoothly.

This work explored the effects and mechanisms of MSI2 on radiosensitivity of lung cancer cells, including the confirmation of MSI2 on radiosensitivity, the regulatory role on DNA damage repair, and the key targets and interrelationships. We further elucidated the effects of MSI2 on radiosensitivity of lung cancer cells and its patterns, clarified the regulatory role of MSI2 on DNA damage repair in lung cancer cells, and revealed the molecular mechanism of MSI2 on radiosensitivity of lung cancer cells. This study will not only help to elucidate the molecular mechanism of resistance to lung cancer radiotherapy, but also provide new targets and new ideas to improve the effectiveness of lung cancer radiotherapy.

## 4 MATERIALS AND METHODS

### 4.1 Cell culture and treatment

Human embryonic renal epithelial cell line 293T, human pulmonary bronchial epithelial cell line BEAS‐2B cells, and human NSCLC cell lines A549, H460, H1299, H358, and H1975 were obtained from the American Type Culture Collection (ATCC) and cultured according to ATCC guidelines. BEAS‐2B cells were cultured with BEpiCM‐b medium. 293T cells were cultured with DMEM (Dulbecco's modified eagle medium) high‐sugar medium. A549, H460, H1299, H358, and H1975 cells were cultured with RPMI (Roswell Park Memorial Institue) 1640 medium. Then, 10% fetal bovine serum and 1% penicillin streptomycin‒glutamine were added to the medium. The cells were maintained at 37°C in a humidified incubator containing 5% CO_2_. Cells requiring irradiation treatment are plated 24 h prior to irradiation, and the inhibitor VE‐821 (Selleck, S8007) was added 2 h before cell irradiation at a final concentration of 10 µM.

### 4.2 Mice and treatments

Nude mice were purchased from the Chinese Academy of Sciences and were 4−5 weeks old. Nude mice were fed with sufficient food and water under ventilated, room temperature, and natural circadian rhythm conditions, and the bedding was changed once every 2−3 days according to the actual situation. Nude mice adapted to the new environment for 1 week after purchase, and the tumors were all located under the skin of the connective tissue at the root of the right thigh. Tumor volume in cm^3^ was determined using the formula (length × width^2^)/2, where length is the longest axis and width is the measurement at right angles to the length. The first group was the Scramble + Mock group, in which the tumor‐bearing cells were A549 cells that were overexpressed with negative control lentivirus Mock after infection with knockdown negative control lentivirus Scramble. The second group was the sh‐MSI2 + Mock group, in which the tumor‐bearing cells were A549 cells infected with lentivirus sh‐MSI2 that knocked down MSI2 and then overexpressed negative control lentivirus Mock. The third group was the sh‐RBM17 + Mock group, in which the tumor‐bearing cells were A549 cells infected with the lentivirus sh‐RBM17 that knocked down RBM17 and then overexpressed negative control lentivirus Mock. The fourth group is the sh‐MSI2 + RBM17 group, in which the tumor‐bearing cells were A549 cells infected with lentivirus sh‐MSI2 that knocked down MSI2 and then infected with lentivirus expressing RBM17. The fifth group is the sh‐RBM17 + MSI2 group, in which the tumor‐bearing cells were A549 cells infected with lentivirus sh‐RBM17 that knocked down RBM17 and then infected with MSI2‐expressing lentivirus, with a total of six mice in each group. Three nude mice in each group was randomly selected for local 15 Gy irradiation after 1 month, and tumor size was measured after 1.5 month. Animal experiments were approved by the Ethics Committee of the Naval Medical University.

### 4.3 Irradiation

We exposed cells and mice to γ‐rays using a ^60^Co irradiator located at the Faculty of Naval Medicine, Second Military Medical University, China. Before local irradiation, 7.5% chloral hydrate was used for anesthesia according to the concentration of 50 mL/kg intraperitoneal administration, and the anesthetized nude mice were put into the mouse irradiation box. The limbs were fixed with clips and paper clips, and the part other than the tumor site at the root of the thigh was covered with a lead plate during irradiation. For in vivo experiments, mice were irradiated with larger doses as the dose distribution in tumor and in cells seeded in the flash is different. Based on our experiments and previous works, we chose 15 Gy for local irradiation at the dose rate of 1 Gy/min.^35^, ^36^ The fixation device was quickly removed after irradiation to resuscitate the nude mice as soon as possible, and they were placed in a rat cage for normal rearing after irradiation. The state of the nude mice was observed daily.

### 4.4 Western blotting and immunoprecipitation

Proteins were extracted with Proteo‐JETTM Mammalian Cell Lysis Reagent (Thermo) according to the manufacturer's instructions. The ProteinExt Mammalian Nuclear and Cytoplasmic Protein Extraction Kit was employed to isolate nuclear and cytoplasmic proteins, following the manufacturer's protocol. Subsequently, the samples underwent analysis via Western blotting using chemiluminescent detection. IP was carried out using the Pierce Co‐Immunoprecipitation Kit as per the manufacturer's instructions. The proteins were separated by SDS‒PAGE (sodium dodecyl sulphate‐polyacrylamide gel electrophoresis) and subsequently transferred onto a PVDF (polyvinylidene fluoride) membrane (Millipore). After blocking with 5% non‐fat milk, the membrane was incubated overnight at 4°C with primary antibodies on a shaking table. Primary antibodies included ATR (Abcam, ab2905), p‐ATR (Abcam, ab178407), ATM (Abcam, ab201022), p‐ATM (Abcam, ab36810), CHK1 (Abcam, ab32531), p‐CHK1 (Abcam, ab79758), CHK2 (Abcam, ab109413), p‐CHK2 (Abcam, ab85743), H2AX (Abcam, ab229914), γ‐H2AX (Abcam, ab81299), β‐tubulin (Proteintech, 10068‐1‐AP), MSI2 (Abcam, ab76148), and RBM17 (Abcam, ab204333). Subsequently, the blots were exposed to a secondary antibody, HRP (horseradish peroxidase)‐conjugated immunoglobulin G (Beyotime, diluted at 1:5000), for 1 h at room temperature. Specific protein bands were visualized using Amersham ECL (Millipore) and detected with an Image Quant LAS4000 system (GE Healthcare Life Sciences). Densitometric analysis of protein expression was conducted using ImageJ software (National Institutes of Health).

### 4.5 Tissue chip immunohistochemistry

Eighty cases of lung cancer tissue samples and 80 cases of corresponding paracancerous tissues, a total of 160 tissue samples from Shanghai Pulmonary Hospital, were made into paraffin tissue chips with a diameter of 1.5 mm for each sample, including the marked sites, a total of 161 points. Tissues were stained with antibodies for MSI2. The tissue chip is scanned by a Pannoramic MIDI scanner. After scanning the entire chip content, the Quant center software is used for analysis, and quantitative scoring is carried out according to the color depth, that is, the immunohistochemical score (*H*‐score) score.

### 4.6 Clonogenic assay

Cells in the logarithmic growth phase were harvested, treated with a suitable digestion solution, and then seeded in six‐well plates at an optimal density. For most of the cell experiments, cells were irradiated with 8 Gy based on cell viability assay. For clonogenic assay, after optimization of the radiation doses, cells were irradiated with different doses of ^60^Co (0, 2, 4, and 6 Gy) with three replicates for each group. As cells with MSI2 knockdown could not form clear colonies after 8 Gy irradiation, they were cultured on the 14th day after IR. Subsequently, cells were washed with phosphate‐buffered saline (PBS), fixed with 4% paraformaldehyde, and stained with crystal violet staining solution (Beyotime) for 20 min. Finally, plates were gently washed with water and air‐dried, and colonies containing more than 50 cells were enumerated.

### 4.7 Cell proliferation assay

Cell proliferation was assessed using the Cell Counting Kit‐8 (CCK‐8) (Dojindo). Specifically, cells were suspended and seeded into 96‐well plates at a density of 5 × 10^3^ cells per well. After 48 h of irradiation, cell proliferation was evaluated using the CCK‐8 assay.

### 4.8 Apoptosis analysis

We utilized the Annexin V‐APC/propidium iodide (PI) Apoptosis Detection Kit (BD Pharmingen) to investigate cell apoptosis following the manufacturer's guidelines. Briefly, cell supernatants were collected 24 h post‐irradiation. The cells were detached using trypsin (without EDTA (ethylenediaminetetraacetic acid)) and combined with the supernatant. After centrifugation, cells were washed thrice with PBS and then resuspended in binding buffer. Annexin V and PI were added as per the manufacturer's instructions, followed by a 5‐min incubation in the dark at room temperature. Subsequently, flow cytometry (Beckman) was employed for cell analysis.

### 4.9 Immunofluorescence staining

Cells were cultured on 22 × 22 mm^2^ cover slips in six‐well plates, then subjected to irradiation and fixed with 4% paraformaldehyde for 30 min at specified time points. Subsequently, the cells were permeabilized using a 0.5% Triton X‐100 buffer, followed by blocking with 1% BSA (bovine serum albumin) for 1 h at room temperature. The cells were then incubated overnight at 4°C with primary antibodies against γ‐H2AX (Ser139) (Abcam, ab81299), MSI2 (Abcam, ab76148), and RBM17 (Abcam, ab204333). After washing twice with PBS, the cells were treated with FITC (fluorescein isothiocyanate)‐labeled anti‐mouse antibody (Abcam) or Texas Red‐labeled anti‐rabbit antibody (Abcam) at room temperature for 2 h. Nuclei were stained with DAPI (4,6‐diamino‐2‐phenyl indole) for 15 min in the dark. Confocal microscopy (Zesis 880) with the NIS‐Elements Viewer 4.20 capture system was used to capture images. Six slices of each specimen were observed, and five high‐power visual fields were randomly selected. Protein co‐localization analysis was conducted using ImageJ software, analyzing images of MSI2 and RBM17 in 100 nuclei from three independent experiments. The Pearson correlation coefficient and overlap coefficient were used as statistical measures to quantify co‐localization, with measurements performed using the ImageJ plugin Colocalization Finder.

### 4.10 Lentivirus packaging and stable cell line construction

To prepare lentivirus, 293T cells were cultured in a 10 cm dish until reaching logarithmic growth phase with a cell density of 6−10 × 10^6^ cells. Following the protocol provided by the Lentivirus Packaging Kit (ZR‐LPK‐001, ZORIN Biotech.), plasmids of MSI2 knockdown (sh‐MSI2, GAATGAAGATGTTGTGGAGAA), MSI2 overexpression, RBM17 knockdown (sh‐RBM17, GATGAAGCAGTACGGATATTT), and RBM17 overexpression were synthesized by Shanghai ZORIN Biological Technology Co., Ltd. and transfected into the 293T cells. After transfection, lentiviral supernatants were collected at 24, 48, and 72 h post‐transfection. The collected supernatants were centrifuged at 3500 rpm/min for 10 min and filtered through a 0.45 µm filter. Once the cell density of the logarithmically growing cells reached 60%−70%, the lentiviral supernatant was added to A549 cells. Puromycin (2 mg/mL) was added for selection, and the cells that survived for 96 h were considered stable strains.

### 4.11 Neutral comet assay

DNA double‐strand breaks were assessed by refining the aforementioned neutral comet assay. Initially, slides were immersed in a 1% normal melting agarose solution and allowed to dry thoroughly. Subsequently, a single‐cell suspension was prepared at a concentration of 2 × 10^4^ cells/mL and embedded in low melting agarose under a 40°C water bath. After thorough mixing, the cell suspension was swiftly pipetted onto the surface of the pre‐coated slide. The slides were then incubated at 4°C for 25 min at 25 V in TBE (Tris‐borate‐EDTA) buffer. Following incubation, the gel was stained with PI at a concentration of 10 µg/mL for 20 min and rinsed gently with ddH_2_O. Finally, all gels were examined using an Olympus BX60 fluorescence microscope. A total of 100 images from each slide were analyzed using CASP 1.2.3b2 software (CASPlab).

### 4.12 IP‐MS

After sample processing, mass spectrometry data were acquired using a Q Exactive Plus mass spectrometer (Thermo Scientific) coupled with an EASY‐nLC 1200 liquid chromatography/mass spectrometry system (Thermo Scientific). MaxQuant (V1.6.6) software was employed for searching the mass spectral data, and the Andromeda database search algorithm was applied to explore the Human Proteome Reference Database in UniProt.

### 4.13 Statistical analysis

The data are presented as mean ± SEM for each independent experiment. Statistical analyses were performed using GraphPad Prism 8 software (GraphPad Software Inc.). One‐way analysis of variance followed by the Newman‒Keuls multiple comparison test was used to compare multiple groups, while Student's two‐tailed unpaired *t*‐test was employed to compare differences between two groups. A *p*‐value of <0.05 was considered statistically significant. All experiments were conducted with a minimum of three independent replicates. Graphs were generated using GraphPad Prism 8 software.

## AUTHOR CONTRIBUTIONS

B.L., Y.Y., and B.W. conceived, raised funds for, and supervised the overall project. H.Q. and X.S. carried out the animal experiments. H.Q. performed cell culture experiments. H.Q. and Y.X. drafted the initial version of the manuscript. H.Q. collected and provided clinical samples of lung cancer. H.Q., J.L., S.C., and J.Z. verified the data, images, and text. B.L., H.Q., Y.Y., and B.W. critically revised the work. All authors have edited, read, and approved the final manuscript.

## CONFLICT OF INTEREST STATEMENT

All authors declare they have no conflicts of interest.

## ETHICS STATEMENT

The whole study design and protocols were approved by the Ethics Committee of Naval Medical University, China (approval number: 20200315027).

## Supporting information

Supporting Information

## Data Availability

All data relevant to the study are included in the article or uploaded as Supporting Information.
