# Supplementary material for: Mechanism of Musashi2 affecting radiosensitivity of lung cancer by modulating DNA damage repair
Source: MedComm (2020). 2024 Apr 21;5(5):e548. doi: 10.1002/mco2.548 (PMC11032739; doi:10.1002/mco2.548)
Supplement: Supplementary file 1 — Supporting Information [file MCO2-5-e548-s001.docx]

**Supplementary information**

**Mechanism of MSI2 affecting radiosensitivity of lung cancer through modulating DNA damage repair**

Hongjin Qu^1,2,#^, Xiong Shi^3,#^, Ying Xu^1,#^, Hongran Qin^4^, Junshi Li^1^, Shanlin Cai^1^, Jianpeng Zhao^1^, Bingbing Wan^2,*^, Yanyong Yang^1,*^, Bailong Li^1,*^

^1^Department of Radiation Medicine, Faculty of Naval Medicine, Naval Medical University, 800, Xiangyin Road, 200433, Shanghai, China.

^2^Key Laboratory of Systems Biomedicine (Ministry of Education), Shanghai Center for Systems Biomedicine, Shanghai Jiao Tong University, 200240, Shanghai, China.

^3^Department of Radiology, Stomatological Hospital and Dental School of Tongji University, Shanghai Engineering Research Center of Tooth Restoration and Regeneration, Shanghai, China.

^4^Department of Nuclear Radiation, Shanghai Pulmonary Hospital, School of Medicine, Tongji University, Shanghai, China.

^#^These authors contributed equally: Hongjin Qu, Xiong Shi, Ying Xu.

^*^Corresponding author: Bailong Li, Yanyong Yang, Bingbing Wan

E-mail: libailong2013@163.com, yyyang2010@163.com, wanb@sjtu.edu.cn


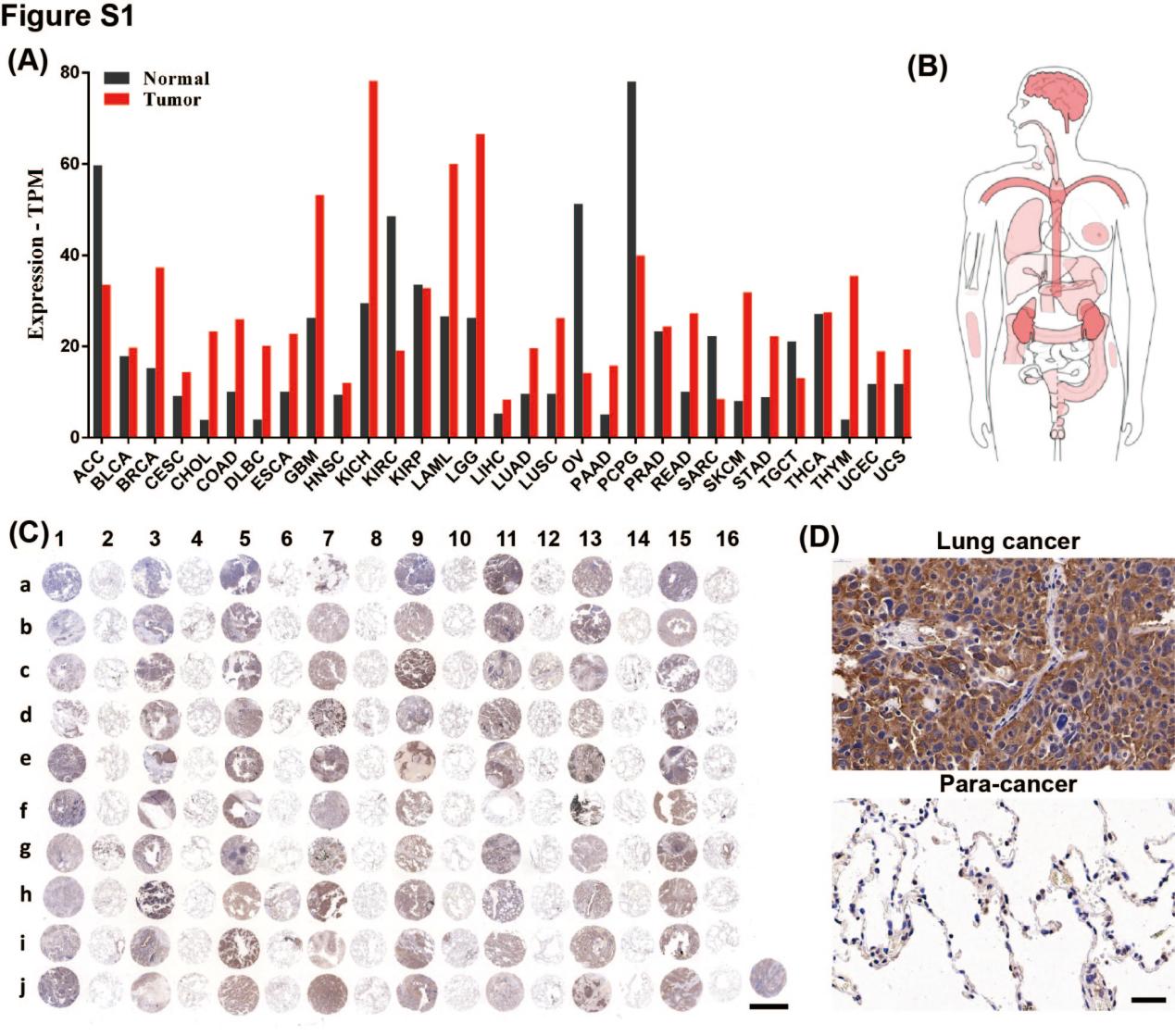


**Supplementary Figure 1.** (A) The gene expression profile of MSI2 across all tumor samples and paired normal tissues. The height of bar represents the median expression of certain tumor type or normal tissue. (B) The expression of MSI2 in tumor samples in bodymap, with darker color representing high expression. Bioinformatics analysis data of (A) and (B) from GEPIA 2. (C) Tissue chip immunohistochemistry detection of lung cancer samples from 80 lung cancer patients, where odd columns are lung cancer tissue samples and even columns are paraneoplastic tissue samples corresponding to the previous odd patients, the sample in the lower right corner is the marker locus, the scale bar represents 1500 μm. (D) Immunohistochemical photographs of sample c9 and sample c10 in (C). Sample c9 is lung cancer tissue and sample c10 is paraneoplastic tissue from the same lung cancer patient. Scale bar represents 20 μm.


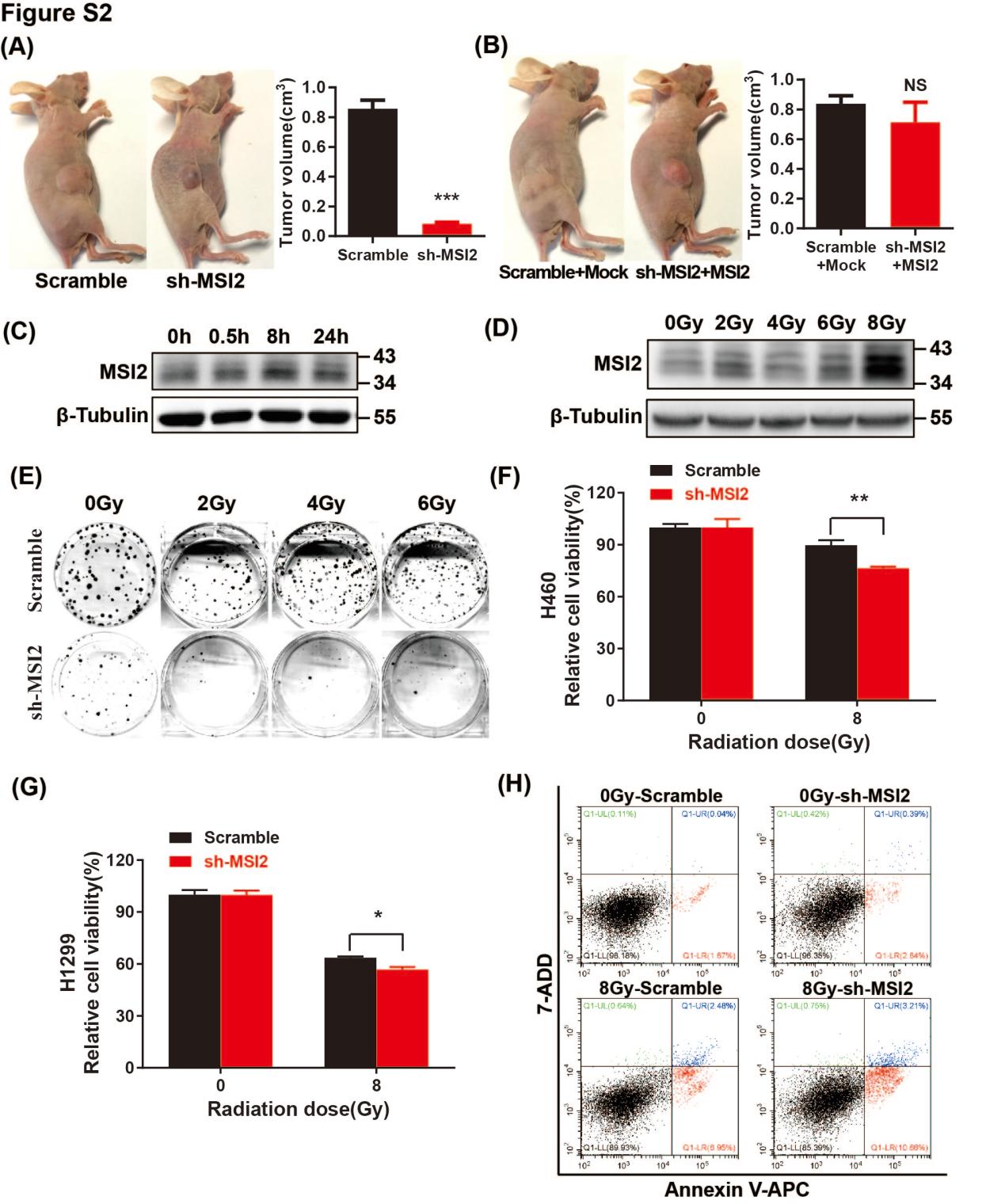


**Supplementary Figure 2.** (A) Photograph of tumor growth and data statistics of tumor volume after knockdown of MSI2 (***P<0.001). (B) Photograph of tumor growth and data statistics of tumor volume after partial restoration of MSI2 expression (***P<0.001). (C) Western blotting tests of MSI2 protein levels at different times after 8Gy irradiation. (D) Western blotting tests of MSI2 protein levels with different radiation doses at 8h. (E) Representative images of clone formation after knockdown of MSI2 at different doses of irradiation. (F) Proliferation level of 8Gy irradiated H460 cells after knockdown of MSI2 (**P<0.01). (G) Proliferation level of 8Gy irradiated H1299 cells after knockdown of MSI2 (*P<0.05). (H) Apoptosis level of 8Gy irradiated A549 cells after knockdown of MSI2.


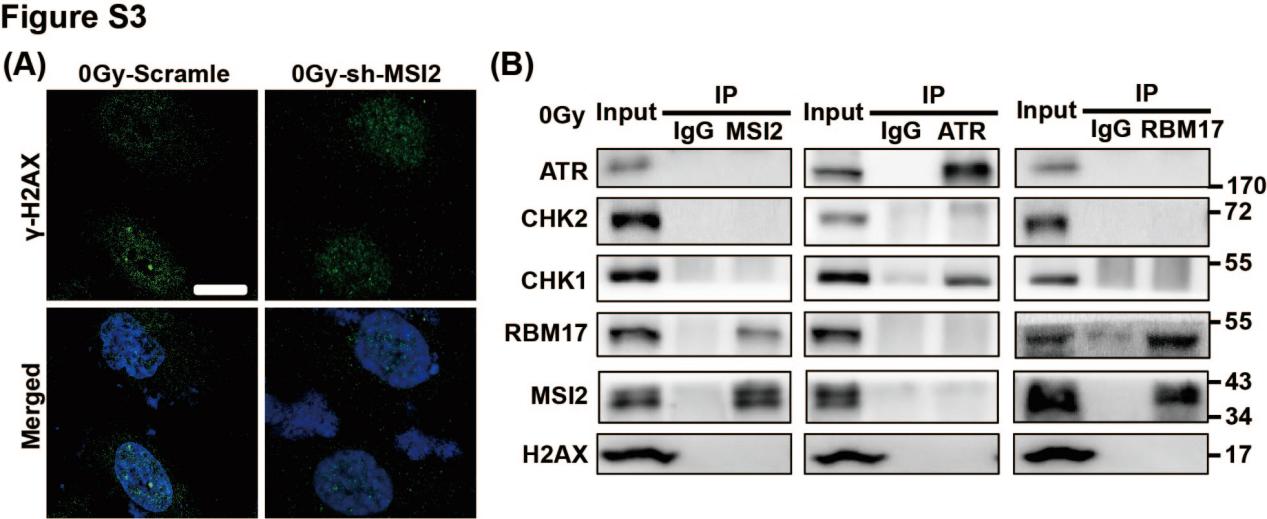


**Supplementary Figure 3.** (A) Representative images of γ-H2AX foci in A549 cells with 0Gy irradiation (scale bar, 10 μm). (B) Immunoprecipitation assay of MSI2, ATR and RBM17 in A549 cells with 0Gy irradiation.


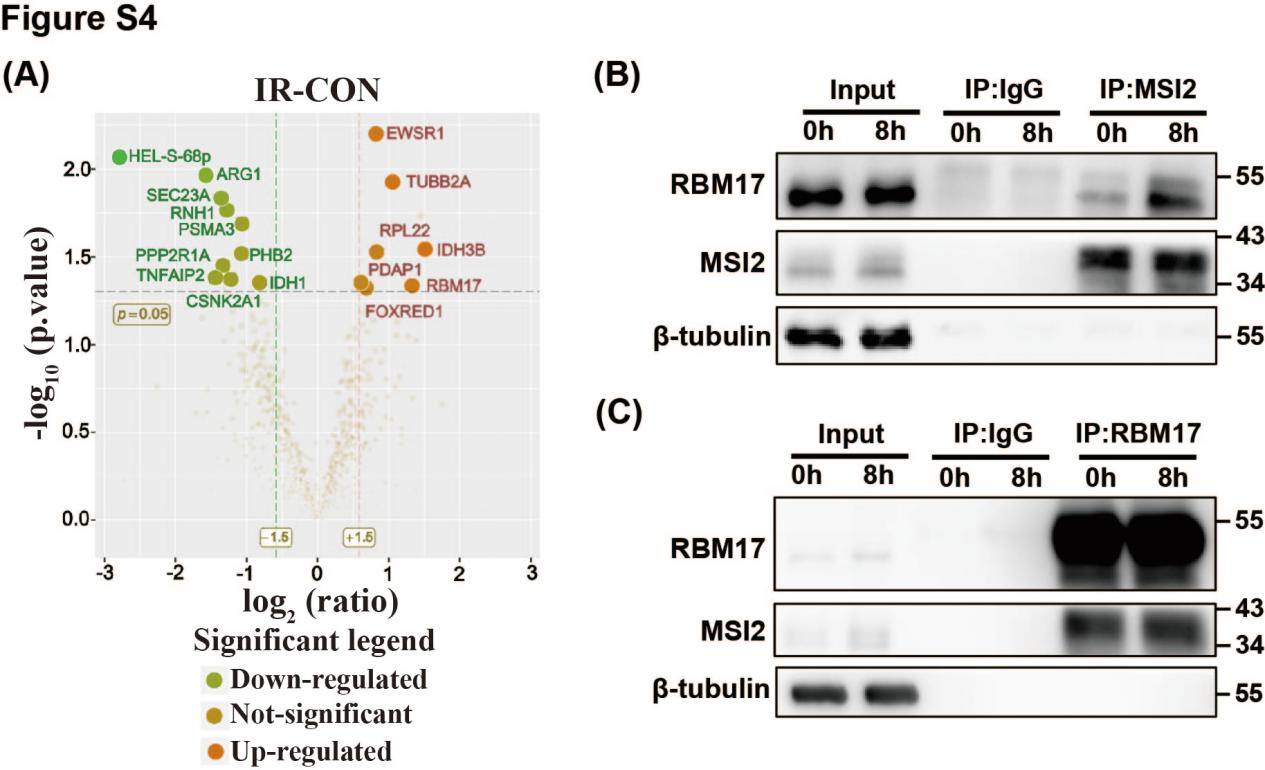


**Supplementary Figure 4.** (A) Volcano plot of bioinformatic analysis of differential proteins in the IP-MS assay. The proteins with significant differences between two groups are shown in dark green (down-regulated) and orange (up-regulated), respectively, and the proteins without statistical differences are shown in light yellow. (B) Immunoprecipitation of RBM17 by using the antibody of MSI2. (C) Immunoprecipitation of MSI2 by using the antibody of RBM17.


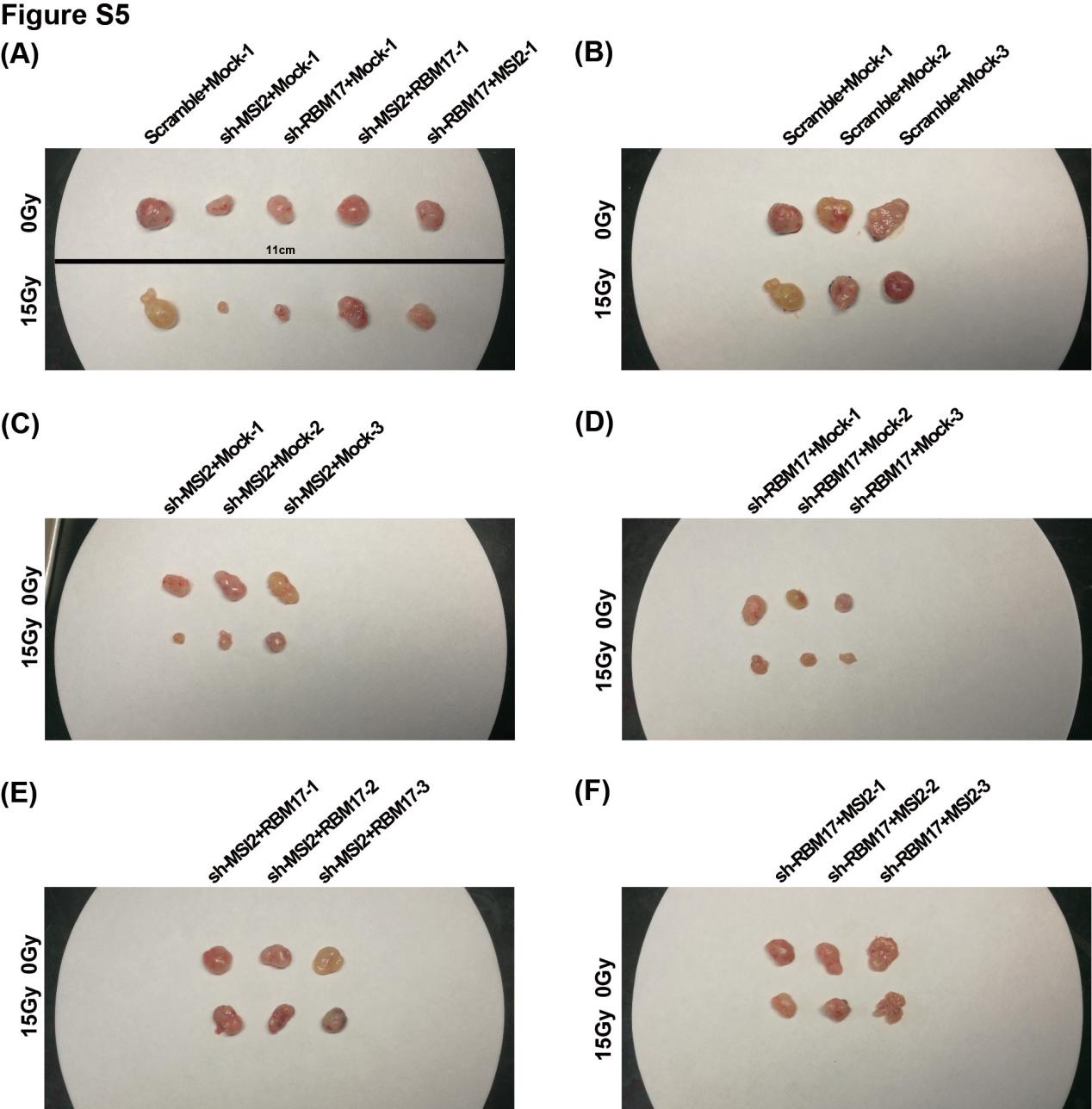


**Supplementary Figure 5.** (A) Photographs of representative tumor bulk specimens of each group in nude mice. (B-F) Photographs of tumor bulk specimens of each group in nude mice from 3 independent experiments. All the photographs of tumors were taken on the same filter paper with a diameter of 11cm.
